# Field Emission Properties of Polymer Graphite Tips Prepared by Membrane Electrochemical Etching

**DOI:** 10.3390/nano10071294

**Published:** 2020-07-01

**Authors:** Alexandr Knápek, Rashid Dallaev, Daniel Burda, Dinara Sobola, Mohammad M. Allaham, Miroslav Horáček, Pavel Kaspar, Milan Matějka, Marwan S. Mousa

**Affiliations:** 1 Institute of Scientific Instruments of the Czech Academy of Sciences, Královopolská 147, 612 64 Brno, Czech Republic; burda@isibrno.cz (D.B.); mih@isibrno.cz (M.H.); mmatejka@isibrno.cz (M.M.); 2 Department of Physics, Faculty of Electrical Engineering and Communication, Brno University of Technology, Technická 2848/8, 616 00 Brno, Czech Republic; xdalla03@vutbr.cz (R.D.); sobola@vutbr.cz (D.S.); kasparp@feec.vutbr.cz (P.K.); 3 Central European Institute of Technology BUT, Purkyňova 123, 612 00 Brno, Czech Republic; 4 Surface Physics and Materials Technology lab, Department of Physics, Mutah University, Al-Karak 61710, Jordan; quantum.master@live.com (M.M.A.); mmousa@mutah.edu.jo (M.S.M.)

**Keywords:** polymer graphite tip, electrochemical etching, field emission microscopy

## Abstract

This paper investigates field emission behavior from the surface of a tip that was prepared from polymer graphite nanocomposites subjected to electrochemical etching. The essence of the tip preparation is to create a membrane of etchant over an electrode metal ring. The graphite rod acts here as an anode and immerses into the membrane filled with alkali etchant. After the etching process, the tip is cleaned and analyzed by Raman spectroscopy, investigating the chemical composition of the tip. The topography information is obtained using the Scanning Electron Microscopy and by Field Emission Microscopy. The evaluation and characterization of field emission behavior is performed at ultra-high vacuum conditions using the Field Emission Microscopy where both the field electron emission pattern projected on the screen and current–voltage characteristics are recorded. The latter is an essential tool that is used both for the imaging of the tip surfaces by electrons that are emitted toward the screen, as well as a tool for measuring current–voltage characteristics that are the input to test field emission orthodoxy.

## 1. Introduction

Graphite materials find their applications in modern electronics with a rapidly increasing frequency [1,2,3,4,5]. They are employed in various sorts of batteries (e.g., graphene anodes), thermocouples, capacitors, transistors, electrical switches, as well as in ion-implantation that is a process that undergirds the production process of present microchips [6,7]. Graphite-based materials such as graphene are also capable of replacing conventional capacitors as its electrical characteristics 10–100 times exceed those of the currently used material—silicon (100 times thinner, 20 times more powerful, significantly superior electrical conductivity, etc.). Graphite-related carbon allotropes are also often incorporated as a source of field emission current in vacuum [8,9,10] or as a source of tunneling current operating both in vacuum and in atmospheric conditions [11].

Polymer graphite (PG) is a relatively young nanocomposite material that was invented mainly for micro-pencil refills containing a polymer-based binding agent and graphite flakes [11]. The electrochemical method that is used by us to obtain a sharp tip is one of the three previously used methods enabling to form the PG rods [11]; however, for the PG, the method has not been studied in more detail yet. The PG has high conductivity and immunity against surface contamination, with a low price, which make it seem a highly suitable material for electrode manufacture in general [11,12,13,14]. Such pointed graphite rods may find various applications in analytical methods; for example, they can be used as a source of free electrons [15] or they can operate with a tunneling current in Scanning Probe Microscopy (SPM) conductive modes [11]. To conclude, it should be mentioned that graphite structures attract the attention of researchers from all over the world. Currently, according to the ScienceDirect, there are almost 1800 articles featuring graphite structures, which speaks in favor of the relevancy of our research. 

This paper continues in this trend, putting as its main goal the characterization of the field emission properties of a PG in correlation to the electrochemical membrane etching method that is used for its preparation. As for the secondary targets, the first one is to provide a comprehensive analysis of a surface on the top of the tip apex achieved mainly by Scanning Electron Microscopy (SEM) and partly by Field Emission Microscopy (FEM). The second one is to describe the electrochemical preparation method and to examine the resultant tip by Raman Spectroscopy (RS). For the RS, the main task is to examine if the final tip is influenced by the type of the PG rod, by the type of etchant and its concentration, or by the etching voltage. To achieve this target, it has been experimented with pencils of different hardness, which is directly connected with the level of clay admixtures added to the graphite during the manufacture. The rods have been etched in alkali solutions—namely in potassium hydroxide (KOH) and sodium hydroxide (NaOH) of different concentrations. 

As for the desirable result, we consider a conical-shaped graphite tip with a minimum amount of impurities, also containing multiple graphite flakes oriented perpendicularly to the tip surface. Therein lies the field emission properties that may appear even for a blunt tip. The number of particular FE sources is the main difference compared to the tip presented in [15], which has been formed by focused ion beam and behaves more similar to a single-source metallic emitter.

## 2. Materials and Methods

### 2.1. Materials

The graphite pencil rods have generally two main constituents: graphite itself and various clay minerals or rocks, which are finely ground (small amounts of wax or polymers are also present to serve as a binder) [11,14]. It was reported that polymer graphite contains usually from 30% up to 80% of sp^3^ hybridized carbon which may shift its properties more toward those of diamond-like carbon (DLC) structures [11]. The macroscopic hardness of polymer graphite rod is determined by the graphite–clay ratio. An increase in the amount of clay will result in a harder rod that is therefore less smudgy when used on paper, whereas by reducing the amount of clay, a softer, more graphite-rich rod may be produced [16]. The conventional pencils are categorized by manufacturers in terms of hardness using different arbitrary grading scales. In this paper, we will be following the European system, which arranges the pencil graphite rods by their hardness in similar manner (from the hardest to the softest): 4H, 3H, 2H, H, F, HB, B, 2B, 3B, 4B, and 5B, etc. [17]. For our experiments, we have used Koh-i-Noor 0.5 micro-pencil leads (KOH-I-NOOR HARDTMUTH, Czech Republic).

### 2.2. Equipment and Methods of Analysis

Raman spectroscopy was utilized to investigate the chemical composition of the tips, whilst the topography of the etched PG tip was obtained using the Scanning Electron Microscopy (SEM) technique. The Raman measurements were obtained using the WITec confocal Raman imaging system utilizing green laser (532 nm, 30 mW) and the exposure of 10 × 10 s with a 50 × objective. The Raman spectra were evaluated and fitted with Lorentzian peak shapes using Fityk 1.3.1 software (Marcin Wojdyr, Warsaw, Poland). The SEM measurements were carried out in Tescan Lyra3 scanning electron microscope with an in-built Aztec SSD detector (Oxford Instruments). For the etching, a modified version of an NT-MDT Solver Nano etching device has been used. The field emission microscope (FEM) that was used to describe tip electrical properties is a custom-made device designed by Horáček and Knápek in the Institute of Scientific Instruments of the Czech Academy of Sciences.

#### Raman Spectroscopy

To obtain additional information about the composition of etched polymer graphite tips, broad range spectra from 100 cm^−1^ to 3500 cm^−1^ have been recorded. Raman spectroscopy is a powerful analytic tool that has been widely used in geosciences. Raman spectra of clay minerals are well documented, and the main peaks usually lie in the 3750–3550 cm^−1^ region attributed to various OH group stretching modes (such as Al-OH, Mg-OH), and in the 0–1200 cm^−1^ region, they are attributed mainly to O–Si–O and O–A1–O, Si–O–Si groups and various OH group bending modes [18,19]. The mass fraction of clay content in polymer graphite rods is generally high; looking at other polymer graphite manufacturers, it is around 20% and 50% for grade 2B and H hardness, respectively [20]. Despite the high mass fraction of clays in the final polymer graphite rods, the Raman bands from the region of 0–1200 cm^−1^, which are attributed to the presence of clays and clay minerals in samples, were not visible in any of the recorded spectra.

Unsurprisingly, the main carbon Raman peaks are well visible. It can be seen that the two most intensive Raman peaks in our scenario occur in the region between 1000 and 1600 cm^−1^. The first peak at 1345–1350 cm^−1^ is associated with breathing modes and disorders of graphene sp^2^ rings (D-band). The peak at 1575–1580 cm^−1^ belongs to the G-band, which is associated with the stretching of all pairs of sp^2^ atoms [21]. The second region 2400–3300 cm^−1^ contains a 2D band at 2710 cm^−1^ and a less intensive G*-band at 2445 cm^−1^ and 2D’-band at 3240 cm^−1^. Among these, the 2D-band at around 2700 cm^−1^ gives information about the turbostraticity of graphite; it is undivided in the case of highly oriented graphite, but in the case of structured graphite or amorphous carbon, the 2D-band is significantly broader.

**Figure 1:**
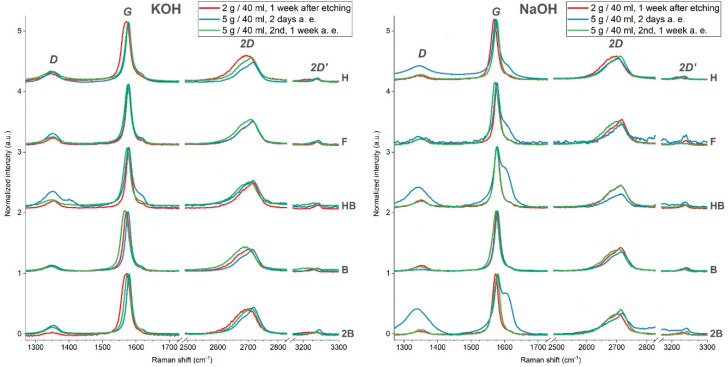
Comparison of Raman spectra of polymer graphite rods of different hardness etched: in 2 g/40 mL (one set) and 5 g/40 mL KOH solution; two sets of experiments (**left**) and secondly (**right**), they were etched in 2 g/40 mL (one set) and 5 g/40 mL NaOH solution. Raman spectra were captured two days or one week after etching, as indicated.

Raman spectra of etched polymer graphite rods in the graphite range can be seen in Figure 1. The spectra are normalized by dividing each of them by the intensity value at the maximum of its G-band. Three sets of measurements have been carried out with both NaOH and KOH etching solutions. When comparing etchants of the same concentration between each other, the overall peak shapes and positions vary only slightly, with the exception of the first sets with 5 g/40 mL concentration (marked blue). Those sets were analyzed with Raman spectroscopy just two days after the etching, whereas the rest of the etched tips were analyzed later, after one week (red and green lines). The spectra measured after 1 week also do not seem to be influenced by the choice of KOH versus NaOH. Even when comparing spectra by the hardness from the same set, there are just slight differences. Based on the Raman spectra, those samples can be categorized as macroscopic-size graphite flakes.

Only the tips analyzed after two days seem to be unlike others, namely 2B, HB, F, H from 5 g NaOH/40 mL. The D-band of those samples is significantly more intensive; also, the presence of a G-band leg toward higher wavenumbers indicates an increase of defects or the presence of unstable compounds on the etched surface formed during or after the amorphization of graphite during the etching. We suppose this may be a temporary effect, as these features were not visible when measuring the one-week-old etched tips. The absence of a G-band leg in the spectra of samples measured after one week may be related to the degradation or evaporation processes of unstable compounds during the one-week period.

It was found out that the spectra taken at the etched tips of polymer graphite show more crystalline graphite than the unetched side layers of the rods. The amorphization of the side layer graphite flakes most likely happens during the extrusion step of the manufacture of polymer graphite rods. During the tip preparation, the side layer is locally etched away, and more crystalline graphite flakes at the tip are uncovered.

It can be noted that during the etching, the amorphous side layer of the polymer graphite pencil rod, which is probably formed during the extrusion step of manufacture, is etched away, and thus, more crystalline bulk graphite is uncovered after the etching. 

The efforts on the subject considering the material composition of various polymer graphite rods and noise analyses of prepared field electron emitters were published earlier [11,15]. The SPM probes and field electron emitters prepared from polymer graphite pencil leads of various hardness values and from different manufacturers are greatly dissimilar in terms of the tip radius and the performance. The admixtures, which are among the main affecting factors, were shown to be rather complex [11].

### 2.3. Electrochemical Membrane-Etching Method

**Figure 2:**
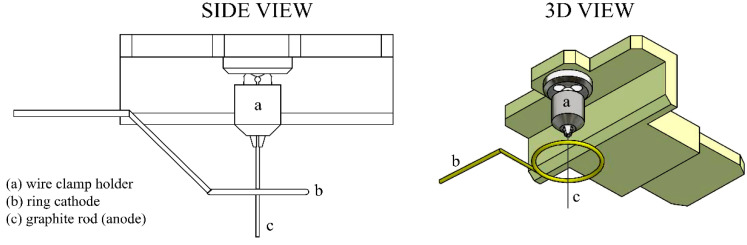
A precise clamp holder extension intended for 0.2–0.4 mm diameter wires’ perpendicular fixation toward the electrolyte surface.

The membrane-etching method that has been used for a sample preparation shares same electronic configuration as a tip-etching method that takes place in the filled cylinder, which was published earlier [22]. Instead of the cylinder, we are working with a thin membrane contained inside the ring cathode illustrated in Figure 2. Left. In addition, the membrane-etching method is based on the well-known drop-off method of etching that was developed to be used originally for single-crystalline tungsten field-emission tips production. The electrochemical method of tip preparation and sharpening provides good reproducibility of the tip shape and sharpness, as it was reported in the same paper [22]. 

The type and concentration of hydroxide (NaOH, KOH) along with the voltage of the etching current allows controlling the tip-etching rate and thus the tip quality. Qualitative parameters are discussed further in the text in more detail.

The potential between the etched rod (anode) and ring that creates a membrane with the alkali electrolyte (cathode) is usually set from 5 to 12 Volts DC. The etching voltage affects the etching speed and linearity of the etching current. This means that for voltages higher than 5 V, the bubbles generation during the etching (on cathode) is more noticeable, resulting in etching current fluctuations and thus affecting the resulting tip regularity and hence the tip reproducibility. The current fluctuations of the etching current are also dependent on the composition of the PG rod. Even for the same brand, each production series has slightly different chemical composition (based on our previous experiences, the difference in composition could be up to 5%). 

In order to provide a precise cone-shaped tip and hence to increase production repeatability, it is desirable to work with 5 V voltage despite the etching time being longer in proportion to the increased etching voltage.

As for the mechanical part of the etching set-up, an etching station for probe production made originally by NT-MDT Company (NT-MDT Spectrum Instruments, Moscow, Russia) was used and partially modified by adding a precise clamp-holder to fix the graphite rod during etching. This allowed a precisely perpendicular attachment of the tip toward the electrolyte surface, as it is illustrated in Figure 2, and hence increasing the symmetry of the produced tip. During the etching, one of the graphite rod’s ends passes through a conducting diaphragm that keeps a drop of alkali solution, providing the necessary surface tension that cuts the rod as follows. As the bottom part falls off due to its own weight, the electric circuit providing the etching current switches off. The main requirement for adjustment of the existing setup for etching and the sharpening of graphite probes was the positioning of the pencil lead perpendicular to the ring electrode.

## 3. Results and Discussion

### 3.1. Description of the Electrochemical Etching

It is not a trivial task to fully represent the whole range of chemical reactions that take place during the electrochemical etching of a graphite pencil lead due to its multicomponent nature. It has been reported [23] that clay minerals are chemically attacked by NaOH, which results in portions of SiO_2_, Al_2_O_3_, MgO, and Fe_2_O_3_, which they contain, to be etched away. Based on our previous ICP-MS analysis of PG rods, the clay minerals may be from a kaolinite group such as kaolinite or illite. However, chemical reactions of clay with hydroxide are very slow [23] compared to the hydroxide etching of graphite. However, we can be quite certain that at a least part of the graphite undergoes the following reactions illustrated by Equations (1)–(6) in a different proportion, since we were able to observe quite an intense emission of gas throughout the process.

C + 4NaOH → 4Na + CO_2_↑ + 2H_2_O (1)

C + 4KOH → 4K + CO_2_↑ + 2H_2_O (2)

C + 2NaOH → 2Na + CO↑ + H_2_O (3)

C + 2KOH → 2K + CO↑ + H_2_O (4)

C + NaOH + H_2_O → H_2_↑ + Na_2_CO_3_ (5)

C + KOH + H_2_O → H_2_↑ + K_2_CO_3_ (6)

**Figure 3:**
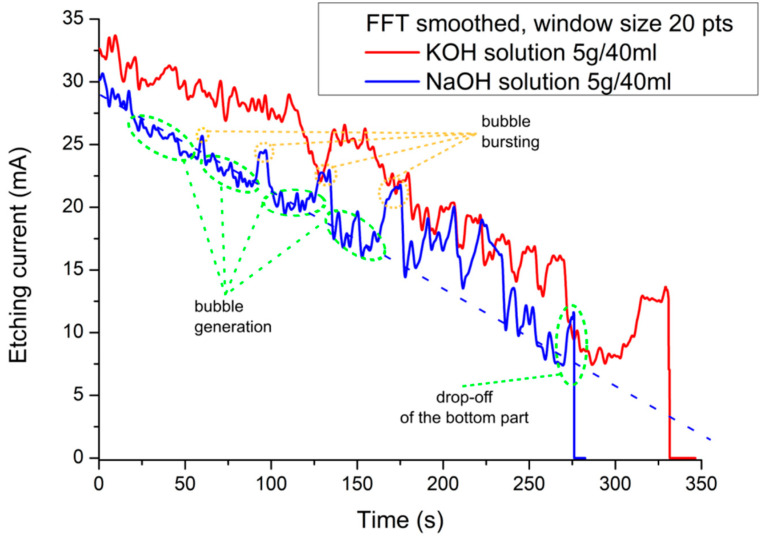
The etching current of a polymer graphite rod using two different etchants.

The bulk of the graphite rod most likely remains unreacted and precipitates on the metal ring in the form of flakes. The gas generation is illustrated in Figure 3 by etching current waveforms. 

There are two different waveforms for two different solutions etched by the same etching voltage (5 V) showing a quasiperiodic occurrence of peaks that are connected to the generation of bubbles. When a bubble is generated near the etched tip, the surface that is in connection with the etchant starts to be reduced, and hence the etching current starts decreasing. When the equilibrium between the air pressure and the surface tension of the bubble is broken, the bubble pops whilst increasing the wetted area of the etched rod and, in this way, it increases the etching current. This effect also appears quasiperiodically. The slope of the etching current versus time is usually linear depending on the conductivity of the bulk.

The end of the etching is initiated by a drop-off of the bottom part of the rod steeply decreasing the etching current, which can be identified by a differential detector [22]. Recently, this technique proved to be very effective as an indicator of the drop-off moment for the etching in a volume of electrolyte (oppositely to the membrane etching), where it is more crucial to disconnect the etching current in time in order to prevent tip blunting [22].

### 3.2. Scanning Electron Microscopy Analysis

**Figure 4:**
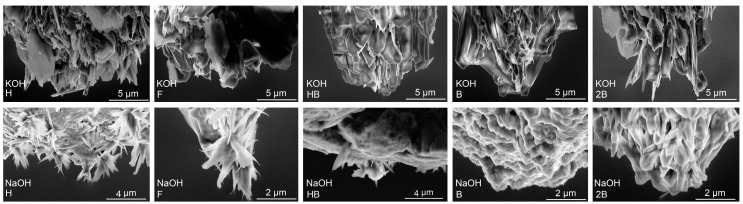
SEM image of the tips of various hardnesses (H, F, HB, B, and 2B) that were produced by electrochemical etching using KOH (**top row**) and NaOH (**bottom row**). The material used for these tips is Koh-i-Noor PG [22].

The initial analysis of the polymer graphite tip, providing basic tip geometry and topography, was obtained by classical Scanning Electron Microscopy. The SEM measurements were done using 5 keV primary beam energy and the Everhart-Thornley Secondary Electrons detector [11]. Those observations have been done on Koh-i-Noor PG rods of various hardnesses going from H, F, HB, B, to 2B etched by KOH and NaOH etchants, as it is illustrated in Figure 4. The top row within the Figure 4 illustrates tips that were created using KOH. The bottom row of the same figure illustrates the NaOH-etched tips. Both lines are divided into five rows showing one hardness. It is shown that that the tips are, from the point of view of geometry, rather blunt (the diameter is in the micrometer-scale rather than nano-scale); however, they contain multiple sharp tips that are more or less oriented perpendicularly against the tip surface plane. These images also demonstrate that the reproducibility of the tip’s surface structure is very low. It can be seen that some of the flakes may have a sufficient field gradient when connected to the negative power supply in the vacuum chamber of the FEM and act as a partial electron source. For this reason, the whole tip is considered more like a Large Area Field Emitter (LAFE) composed of non-homogeneously distributed tips of different sharpness and hence different field enhancement. According to the SEM measurements illustrated in Figure 4, it seems that the surface of the etched rods becomes more sharp for the rods with hardnesses H, F, and partly HB.

Due to the charging effects and beam interaction of the surface, the analysis provided by SEM is limited, even at very low energies (incorporating so-called “breaking voltage”) and a very low primary electron beam current. In addition, this fact made us use the Field Emission Microscopy technique that proved to be an ideal tool to evaluate field emission properties in general.

### 3.3. Field Emission Microscopy Analysis

The Field Emission Microscopy (FEM) was originally invented by Erwin W. Müller, creating an analytical technique suitable for use in materials science to investigate molecular surface structures and their electronic properties [24]. In the last five decades, the methods of interpretation of the current–voltage data provided by measuring the total emission current in FEM have been considerable improved and extended mainly by Richard G. Forbes [25], on whose work we base our interpretation of the experimentally obtained I-V data. With consideration to our recent work [11], where polymer graphite tips were suggested to be used as a cheap STM probe, the use of the FEM for tip analysis makes here very good sense, making use of the field emission properties of the materials.

**Figure 5:**
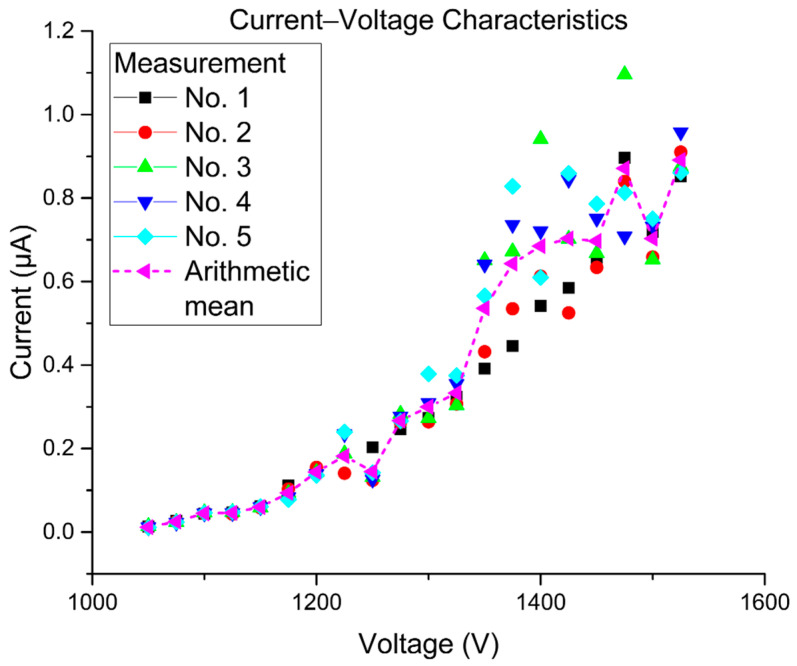
Current–voltage characteristics were recorded for 5 cycles.

The structure located on the surface has been put in a FEM, inside a strong electric field, whose electric potential was set above the threshold voltage (V > 650 Volts), where the total emission current collected by a scintillator started following the Fowler–Nordheim principles, and so the PG etched tip behaved similar to a field-emission cathode. Experimental data have been obtained in the field emission region that is located above the tunneling current region, and the potential was therefore set approximately above 1000 Volts and measured in steps of 25 V up to 2000 Volts. The effect of the limiting resistor, which is connected serially to protect the cathode tip, started to be apparent approximately above 1700 Volts where the current that was measured by a conductive aluminum layer on the Cerium doped Yttrium aluminum garnet (YAG: Ce) showed currents higher than 5 microamperes. It should not be omitted that the collection efficiency of the scintillator used is approximately 80% comparing to a Faraday cage. Therefore, this setback introduces a systematic error where all our values are measured lower than they really are. Figure 5 presents the I-V characteristics of a polymer graphite electron emitter in diode configuration that was measured in the field electron microscope equipped with a pair of Varian Vaclon Plus 55 Starcell ion pumps.

The emission current between the emitter and Al-coated scintillator was measured by a precise Agilent 33410a digital multimeter enabling extended measurement functions. In order to compensate for the current fluctuations, for each measurement located in between 1 and 2 kV, an integration feature was used. The threshold voltage was found out to be 1050 V. By further increasing the applied voltage, the emission current increases and starts to fluctuate. This behavior is believed to be directly attributed to the degradation of the graphite structure—to be more specific, the ripping and formation of graphite flakes where the electrons do really escape. To assure the optimal pressure needed for the measurement, the setup has to be conditioned first. The FEM setup was pumped down by an ion pump and baked at 150 °C for 8 h, then left to cool, while still being continuously pumped down. This way, the lower range of the ultra-high vacuum was achieved (10^−7^ Pa) prior to the actual measurement. 

**Figure 6:**
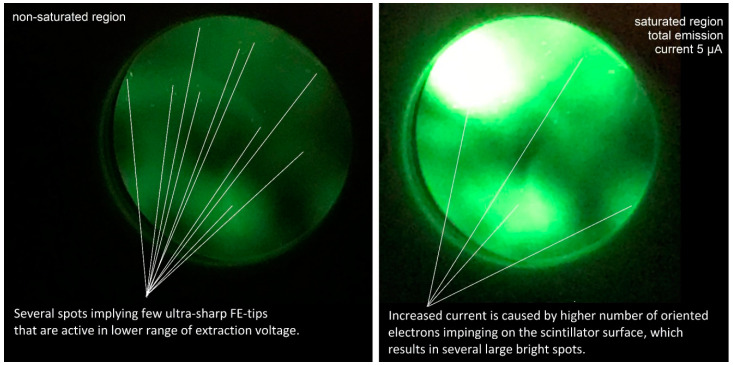
Field Emission Microscope Pattern Images for non-saturated (**left**) and saturated (**right**) region of the I-V plot. The diameter of the scintillator plane here is 31.5 mm.

Figure 6 presents the FEM pattern image which describes the emission total current and its fluctuations. As shown in the figure, the pattern contains bright small dots that describe multiple locations for the orthogonal to the tip surface that provide a higher number of electrons to reach the scintillator screen; these electrons are believed to have higher energies than other electrons, since they were emitted directly to the screen. 

As mentioned earlier, the surface of the tip is considered to be a Large Area Field Emitter (LAFE), and to completely analyze this type of emitter, we need to extract two important characterization parameters which are the Macroscopic Field Enhancement Factor M – FEF (γ_M_) and the Formal Area Efficiency ($\alpha_{f}^{\mathrm{SN}}$) for Schottky–Nordheim (SN) as a potential barrier. In order to do these analyses, the Murphy–Good plots [25] are used to analyze the obtained data and to extract the characterization parameters for the emitter.

**Figure 7:**
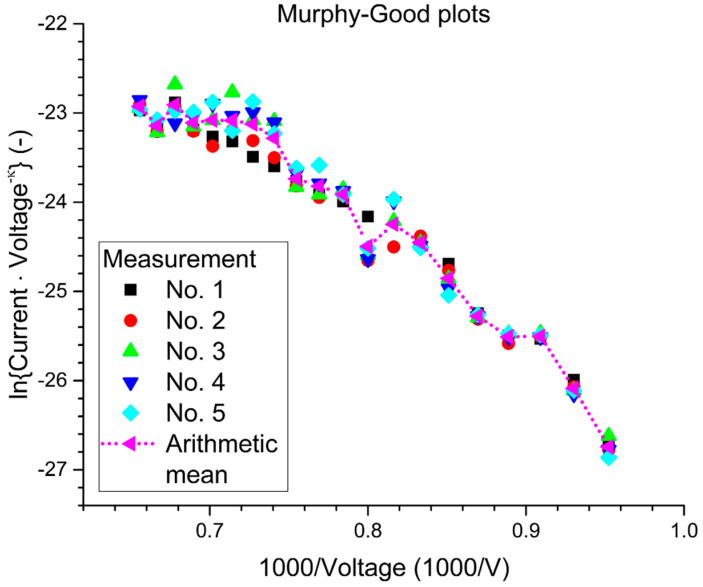
The analysis plots for the five cycles of the tested tip.

**Figure 8:**
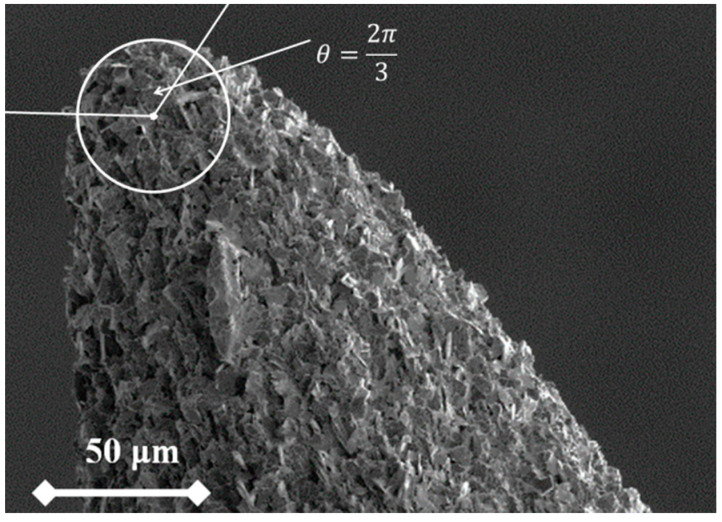
Calculation of the macroscopic area. For the illustration, the HB polymer graphite (PG) rod image was used.

These plots take the form of ln(I/V^κ^) versus 1/V, where *κ* is a function of the work function (*φ*) for the used material (*κ* =1.22719212 for *φ* = 4.5 eV). Figure 7 presents the analysis plots for the 5 periods for the tested tip, and Table 1 presents the extracted data from these plots. Table 1 illustrates the extracted parameters of the emitter from the Murphy–Good plots. Here, the *R* is the apex radius of the emitter, ${\left\{A_{f}^{\mathrm{SN}}\right\}}^{\mathrm{extr}}$ is the extracted formal emission area, $\xi_{C}^{\mathrm{extr}}$ is the extracted characteristic voltage conversion length that describes the effective range of the local electrostatic field at the surface of the tip, ${\left\{\alpha_{f}^{\mathrm{SN}}\right\}}^{\mathrm{extr}}\left[={\left\{A_{f}^{\mathrm{SN}}\right\}}^{\mathrm{extr}}/A_{M}\right]$ is the extracted formal area efficiency, which describes as the ration between the effective emission area and the real macroscopic area of the effective surface, $\gamma_{M}^{\mathrm{extr}}\left[=d_{M}/\xi_{C}^{\mathrm{extr}}\right]$ is the macroscopic field enhancement factor with $d_{M}=1$ mm being the system distance used to define the field enhancement factor, and $A_{M}=1590\;{\mu m}^{2}$ is the large area field emitter macroscopic area. The macroscopic area was calculated by integrating the surface area element of the sphere that is presented in Figure 8, where:

$$A_{M}=R^{2}\int_{0}^{\pi /3}Sin\left[\theta \right]d\theta \int_{0}^{2\pi }d\varphi .$$

## 4. Conclusions

This paper presents the field emission behavior of field emission tips containing multiple graphite flakes that are behaving similar to particular electron sources on a single tip. To obtain a sample, we have adapted a device using a well-established method of electrochemical etching to create a tip from a composite material based on graphite flakes in a membrane of alkali etchant. The electrochemical setup has been described along with particular chemical processes taking place during the process of PG etching. 

Before the analysis of the field emission properties, it was necessary to describe the effect of the electrochemical etching and to evaluate multiple PG nanocomposites that are currently commercially available. The Raman spectroscopy that was used for the evaluation proved that after the etching, there may be a temporal change of surface properties indicated by a strong increase of D-bands and the presence of a G-band leg, which changes after a longer period of one week. Other than that, Raman spectra of graphite of etched tips do not show any conspicuous differences in peak shapes and positions. The Raman spectroscopy bands related to clay minerals are not visible. The topographic information of the tip surface was provided by Scanning Electron Microscopy and Field Emission Microscopy. The SEM showed multiple graphite flakes originated from the PG rod and oriented perpendicularly toward the rod, which has been lately put in a correlation to the bright spots on the FEM screen showing the areas containing particularly emitting surface of the PG tip.

The field emission analysis is based on a finding presented in our previous work [11] reporting favorable properties’ tunneling behavior. For our analysis, we have increased the current density and inter-electrode distance, transferring the work mode to the field emission region, where it is possible to take advantage of the recent methodology developed by Forbes [25,26,27] and hence to fully characterize the tip as for the field-emission point of view.

In order to analyze the field emission properties, the FEM was used to examine the tip surface and to describe the field-emission behavior of graphite flakes present on the tip apex. The analysis is based on current–voltage data measured in the classical FEM using a recent methodology based on Forbes’ Field Emission Orthodoxy Test, which has been used to prove the quantum-tunneling nature of the measured current. This analysis also implied the existence of multiple sources (sharp tips) that are sufficiently sharp to achieve a field gradient strong enough to provide the field emission current. From Table 1, the values of the formal emission area efficiency are in the range of ($\times 10^{-10}$). So, the total effective emission area was much lower than the real macroscopic area, which is in agreement with the hypothesis that the effective emission was from the flakes’ apexes as shown by the bright dots in Figure 6. This is in perfect agreement with the topographical analysis provided by the SEM showing multiple flakes oriented perpendicularly toward the tip surface. The latter arrangement behaves similarly to general LAFE providing extended tunneling capabilities even for a tip of a relatively blunt diameter.

## Figures and Tables

**Table 1:** Extracted emitter’s parameters were obtained from the Murphy–Good plots.

Measure n.	Fitting Points		R [μm]	*Φ* [eV]	Slope [Np.V]	${\left\{A_{f}^{\mathrm{SN}}\right\}}^{\mathrm{extr}}$ [nm^2^]	$\xi_{C}^{\mathrm{extr}}$ [nm]	${\left\{\alpha_{f}^{\mathrm{SN}}\right\}}^{\mathrm{extr}}\times 10^{-10}$	$\gamma_{M}^{\mathrm{extr}}\times 10^{3}$
	x [V^−1^]	y [Np]							
**1**	0.0007	−22.1	22.5	4.5	−17000	1.0350	260.71	6.509625	3.8357
	0.0009	−25.5							
**2**	0.0007	−22.9	22.5	4.5	−13000	0.9096	199.36	5.720596	5.0159
	0.0009	−25.5							
**3**	0.0007	−22.7	22.5	4.5	−14000	1.6424	214.70	10.32977	4.6577
	0.0009	−25.5							
**4**	0.0007	−23.1	22.5	4.5	−12000	1.6112	184.03	10.13346	5.4339
	0.0009	−25.5							
**5**	0.0007	−23	22.5	4.5	−12500	1.7456	191.70	10.97855	5.2166
	0.0009	−25.5							
**Mean**	0.0007	−23	22.5	4.5	−12500	1.2932	191.70	8.133221	5.2166
	0.0009	−25.5							
